# The human amniotic fluid stem cell secretome triggers intracellular Ca^2+^ oscillations, NF‐κB nuclear translocation and tube formation in human endothelial colony‐forming cells

**DOI:** 10.1111/jcmm.16739

**Published:** 2021-07-20

**Authors:** Valentina Balducci, Pawan Faris, Carolina Balbi, Ambra Costa, Sharon Negri, Vittorio Rosti, Sveva Bollini, Francesco Moccia

**Affiliations:** ^1^ Department of Biology and Biotechnology “Lazzaro Spallanzani” Laboratory of General Physiology University of Pavia Pavia Italy; ^2^ Department of Experimental Medicine (DIMES) University of Genova Genova Italy; ^3^ Laboratory of Biochemistry, Biotechnology and Advanced Diagnostic Myelofibrosis Study Centre Fondazione IRCCS Policlinico San Matteo Pavia Italy; ^4^ Present address: Department of NEUROFARBA Center of Molecular Medicine University of Firenze Firenze Italy; ^5^ Present address: Laboratory of Cellular and Molecular Cardiology Cardiocentro Ticino Lugano Switzerland; ^6^ Present address: Center for Molecular Cardiology University of Zurich Zurich Switzerland

**Keywords:** angiogenesis, Ca^2+^ signalling, endothelial colony‐forming cells, human amniotic fluid stem cell secretome, InsP3Rs, NAADP, NF‐κB, paracrine therapy, TRPV4, tubulogenesis

## Abstract

Second trimester foetal human amniotic fluid‐derived stem cells (hAFS) have been shown to possess remarkable cardioprotective paracrine potential in different preclinical models of myocardial injury and drug‐induced cardiotoxicity. The hAFS secretome, namely the total soluble factors released by cells in their conditioned medium (hAFS‐CM), can also strongly sustain in vivo angiogenesis in a murine model of acute myocardial infarction (MI) and stimulates human endothelial colony‐forming cells (ECFCs), the only truly recognized endothelial progenitor, to form capillary‐like structures in vitro. Preliminary work demonstrated that the hypoxic hAFS secretome (hAFS‐CM^Hypo^) triggers intracellular Ca^2+^ oscillations in human ECFCs, but the underlying mechanisms and the downstream Ca^2+^‐dependent effectors remain elusive. Herein, we found that the secretome obtained by hAFS undergoing hypoxic preconditioning induced intracellular Ca^2+^ oscillations by promoting extracellular Ca^2+^ entry through Transient Receptor Potential Vanilloid 4 (TRPV4). TRPV4‐mediated Ca^2+^ entry, in turn, promoted the concerted interplay between inositol‐1,4,5‐trisphosphate‐ and nicotinic acid adenine dinucleotide phosphate‐induced endogenous Ca^2+^ release and store‐operated Ca^2+^ entry (SOCE). hAFS‐CM^Hypo^‐induced intracellular Ca^2+^ oscillations resulted in the nuclear translocation of the Ca^2+^‐sensitive transcription factor p65 NF‐κB. Finally, inhibition of either intracellular Ca^2+^ oscillations or NF‐κB activity prevented hAFS‐CM^Hypo^‐induced ECFC tube formation. These data shed novel light on the molecular mechanisms whereby hAFS‐CM^Hypo^ induces angiogenesis, thus providing useful insights for future therapeutic strategies against ischaemic‐related myocardial injury.

## 1 INTRODUCTION

Endothelial colony‐forming cells (ECFCs) represent vasculogenic cells that are truly committed to the endothelial lineage and, throughout postnatal life, are mobilized upon ischaemic injury to restore the damaged vascular network.^1^, ^2^ ECFCs possess a high clonogenic potential, form capillary‐like structures in in vitro Matrigel tubulogenesis assays, integrate into pre‐existing vasculature and rescue local blood perfusion in murine models of ischaemia.^2^, ^3^ Therefore, ECFCs hold remarkable promise as the most suitable cellular substrate to induce therapeutic angiogenesis in ischaemic disorders, such as acute myocardial infarction (AMI), peripheral artery disease, and stroke.^2^, ^3^, ^4^ Genetic manipulation and pharmacological conditioning could be exploited to improve ECFCs’ angiogenic activity and/or to improve their survival/engraftment within the harsh microenvironment of the ischaemic tissue.^2^, ^5^ Mesenchymal stromal progenitors obtained from leftover samples of second trimester amniotic fluid for prenatal diagnosis have been lately described as appealing therapeutics in several preclinical models of disease. In particular, human amniotic fluid‐derived stem cells (hAFS) have been shown to express a remarkable pro‐angiogenic paracrine potential.^6^, ^7^ A more recent investigation showed that the hAFS secretome collected under hypoxic conditions stimulated circulating ECFCs to assemble into bidimensional capillary‐like networks in vitro through an oscillatory increase in intracellular Ca^2+^ concentration ([Ca^2+^]_i_)^8^, ^9^; furthermore, it actively induced local angiogenesis and promoted cardiac repair in a murine model of AMI.^8^, ^9^ Therefore, the local injection of the hypoxic hAFS secretome could represent an alternative strategy to recruit circulating ECFCs towards the damaged myocardium and induce therapeutic angiogenesis.^2^ The paracrine therapy of AMI would be promptly accessible to the patients and could overcome many of the drawbacks associated with cell‐based therapy, including the time‐consuming procedure and the requirement for huge amounts of cells.^10^, ^11^ Clinical translation of this approach would benefit from the elucidation of the signalling pathways whereby the hAFS secretome triggers the pro‐angiogenic Ca^2+^ response in ECFCs.

Intracellular Ca^2+^ oscillations drive vascular endothelial growth factor (VEGF)‐induced proliferation and tube formation in both circulating^12^ and umbilical cord blood (UCB)‐derived ECFCs.^13^ The oscillatory Ca^2+^ signal elicited by VEGF in ECFCs is shaped by rhythmical inositol‐1,4,5‐trisphosphate (InsP_3_)‐induced Ca^2+^ release from the endoplasmic reticulum (ER) and sustained over time by store‐operated Ca^2+^ entry (SOCE).^12^ Furthermore, nicotinic acid adenine dinucleotide phosphate (NAADP)‐induced Ca^2+^ mobilization from endolysosomal (EL) vesicles through two‐pore channel 1 (TPC1) may contribute to pattern the spiking response.^14^ VEGF‐induced intracellular Ca^2+^ oscillations, in turn, stimulate angiogenesis by inducing the nuclear translocation of the Ca^2+^‐sensitive transcription factor, nuclear factor κB (NF‐κB).^12^ The intracellular Ca^2+^ oscillations induced by the hypoxic hAFS secretome in circulating ECFCs strongly resemble those elicited by VEGF.^9^ However, the same pattern of intracellular Ca^2+^ signalling could be underlain by diverse mechanisms,^15^, ^16^, ^17^ as previously reported for VEGF in different types of ECFCs. For instance, the dynamic interplay between InsP_3_ receptors (InsP_3_Rs) and SOCE is triggered by extracellular Ca^2+^ entry through Transient Receptor Potential Channel 3 (TRPC3) in UCB‐derived, but not circulating, ECFCs.^12^, ^13^ Moreover, the Ca^2+^‐dependent molecular decoder which translates hAFS secretome‐induced intracellular Ca^2+^ oscillations into a pro‐angiogenic output remains elusive. Therefore, in the present investigation, we have characterized for the first time the mechanisms whereby hypoxic hAFS secretome induces pro‐angiogenic intracellular Ca^2+^ oscillations and one of the downstream Ca^2+^‐dependent decoders, that is NF‐κB, in circulating ECFCs.

## 2 MATERIALS AND METHODS

### 2.1 Isolation and cultivation of ECFCs

Blood samples (40 mL) collected in EDTA (ethylenediaminetetraacetic acid)‐containing tubes were obtained from healthy human volunteers aged from 22 to 28 years. The Institution Review Board at “Istituto di Ricovero e Cura a Carattere Scientifico Policlinico San Matteo Foundation” in Pavia approved all the protocols. Informed written consent was obtained according to the Declaration of Helsinki of 1975 as revised in 2008. ECFCs were isolated from circulating mononuclear cells, as described in Supplementary Information and in REF.^18^, ^19^

### 2.2 Isolation and preconditioning of hAFS

Human amniotic fluid‐derived stem cells were isolated from leftover samples of second trimester amniotic fluid obtained by prenatal screening from the Prenatal Diagnosis and Perinatal Medicine Unit, IRCCS San Martino Hospital, and the Fetal and Perinatal Medical and Surgery Unit and Human Genetics Laboratory at IRCCS Istituto Gaslini hospital (Genova, Italy). Informed written consent was obtained from all donors, according to local ethical committee authorization (protocol PR 428REG2015) and in compliance with Helsinki Declaration guidelines. After obtaining adherent amniotic fluid mesenchymal cells, hAFS were isolated by immunomagnetic sorting for c‐KIT expression (CD117 MicroBead Kit, Miltenyi Biotechnology), as previously defined^8^, ^9^, ^11^, ^20^ (PMID: 17206138). c‐KIT^+^ hAFS were then cultured in Minimal Essential Medium (MEM)‐alpha with 15% FBS (Gibco—Thermo Fisher Scientific), 18% Chang B and 2% Chang C Medium (Irvine Scientific) with 1% L‐glutamine and 1% penicillin/streptomycin (Gibco—Thermo Fisher Scientific), at 75% confluency before being used to isolate their secretome.

In order to trigger the hAFS paracrine potential and to enrich their secretome with trophic soluble factors, cells were primed in vitro for 24 hours in serum‐free medium (high glucose Dulbecco's Modified Eagle's Medium, DMEM, with 1% L‐glutamine and 1% penicillin/streptomycin, Gibco—Thermo Fisher Scientific) under normoxic (20% O_2_ and 5% CO_2_ at 37°C) or stimulatory hypoxic (1% O_2_ and 5% CO_2_ at 37°C in a Galaxy® 48 R CO_2_ incubators; Eppendorf) conditions.^8^, ^9^, ^11^, ^20^

### 2.3 hAFS secretome separation and concentration

The total cell secretome, as represented by the cell‐conditioned medium (hAFS‐CM) from either hAFS in control normoxic condition (hAFS‐CM^Normo^) or hypoxic preconditioning (hAFS‐CM^Hypo^), was collected as previously described.^8^, ^9^, ^20^ Briefly, hAFS‐CM formulations were centrifuged to remove cell debris and further concentrated using ultrafiltration membranes with a 3 kDa selective cut‐off (Amicon Ultra‐15; Millipore) at 4°C first at 3000 ×*g* for 90’ and then at 3000 ×*g* for additional 30’. hAFS‐CM protein concentration was assessed by BiCinchoninic Acid (BCA) assay (Gibco—Thermo Fisher Scientific). hAFS‐CM was used for in vitro experiments as 80 mg/mL solution to be added to the cell culture medium as from previous studies.^8^, ^9^

#### 2.3.1 Solutions

Physiological salt solution (PSS) had the following composition (in mmol/L): 150 NaCl, 6 KCl, 1.5 CaCl_2_, 1 MgCl_2_, 10 Glucose and 10 Hepes. In Ca^2+^‐free solution (0Ca^2+^), Ca^2+^ was substituted with 2 mmol/L NaCl, and 0.5 mmol/L EGTA was added. Solutions were titrated to pH 7.4 with NaOH. The osmolality of PSS as measured with an osmometer (Wescor 5500) was 338 mmol/kg.

#### 2.3.2 [Ca^2+^]_i_ measurements

Endothelial colony‐forming cells were loaded with 4 µmol/L fura‐2 acetoxymethyl ester (fura‐2/AM; 1 mmol/L stock in dimethyl sulfoxide) in PSS for 1 hour at room temperature. The details of the Ca^2+^ recording set‐up have been described in REF^9^, ^14^ and are reported in the Supplementary Information. All the experiments were performed at room temperature. All the data have been collected from ECFCs isolated from peripheral blood of at least three healthy volunteers.

#### 2.3.3 Immunofluorescence

Twenty‐four hours before treatment with hAFS‐CM^Hypo^ and the specific blockers of intracellular Ca^2+^ signalling, 6 × 10^4^ ECFCs were plated onto 13 mm coverslips in 24‐well plates. ECFCs were fixed in 4% formaldehyde in PBS for 15 minutes at room temperature, permeabilized for 7 minutes in PBS with 0.1% Triton X‐100 and blocked for 30 minutes in 2% gelatin. Then, primary (incubated for 1 hour at 37°C) and secondary (incubated for 1 hour at room temperature) antibodies were applied in PBS with 2% gelatin. The primary anti‐p65 (NF‐κB subunit) antibody specific for immunocytochemistry (Santa Cruz Biotechnology, catalog no. Sc‐372) was used at 1:50 dilution, whereas the AlexaFluor 488 secondary antibody from Invitrogen (catalog no. A‐21441) was used at 1:200. After washing (3 times for 5 minutes each), nuclei were stained with 40,6‐diamidino‐2‐phenylindole dihydrochloride (DAPI) for 15 minutes at room temperature. Fluorescence images were acquired using a Leica epifluorescence microscope equipped with S Fluor X40/1.3 objective using MetaMorph software.

#### 2.3.4 In vitro tube formation assay

Early passage (P2‐P3) ECFCs were cultured in basal medium EBM‐2 supplemented with 2% FBS in Cultrex (Trevigen)‐coated 96‐well plates, in the absence or in the presence of hAFS‐CM^Hypo^ for 24 hours. Capillary network formation was assessed starting from 4 up to 24 hours later. The angiogenic response was measured by evaluating both dimensional and topological parameters. The length of endothelial tube‐like structures (tubules or TLS), number of polygon structures established by TLS, referred to as meshes and indicative of endothelial cell migration, and number of master junctions were measured from acquired bright‐field pictures by using the Angiogenesis Analyzer plugin of ImageJ (Gilles Carpentier, Faculte’ des Sciences et Technologie, Universite’ Paris Est, Creteil Val de Marne, France).^21^, ^22^ Micrographs were captured by using an Olympus IX71‐inverted microscope (Olympus Europa GmbH) equipped with a CPlan F1 10 ×/0.30 objective. Three different sets of experiments, each performed in duplicate, were carried out. To evaluate the effect of Ca^2+^ signalling, the same protocol was repeated by priming ECFC with hAFS‐CM^Hypo^ in the presence of RN‐1734 (20 μmol/L), a selective blocker of transient receptor potential vanilloid 4 (TRPV4),^22^, ^23^ or thymoquinone (25 μmol/L), a specific NF‐κB inhibitor.^12^

#### 2.3.5 Chemicals

Fura‐2/AM was obtained from Molecular Probes (Molecular Probes Europe BV). YM‐58483/BTP‐2 (BTP‐2; 4‐methyl‐4'‐[3,5‐bis(trifluoromethyl)‐1H‐pyrazol‐1‐yl]‐1,2,3‐thiadiazole‐5‐carboxanilide) was purchased from Tocris Bioscience. Glycyl‐l‐phenylalanine 2‐naphthylamide (GPN) was obtained from Santa Cruz Biotechnology. All the chemicals were of analytical grade and obtained from Sigma Chemical Co.

#### 2.3.6 Statistics

All the data have been collected from ECFCs deriving from at least three distinct donors. Pooled data are given as mean ± SE and statistical significance (*P* < .05) was evaluated by Student's *t* test or one‐way ANOVA followed by the post hoc Dunnett's test as appropriate. Data relative to Ca^2+^ signals are presented as mean ± SE, while the number of cells analysed is indicated in the corresponding bar histograms.

## 3 RESULTS

### 3.1 Extracellular Ca^2+^ entry triggers hAFS‐CM^Hypo^‐induced intracellular Ca^2+^ oscillations in circulating ECFCs

**FIGURE 1:**
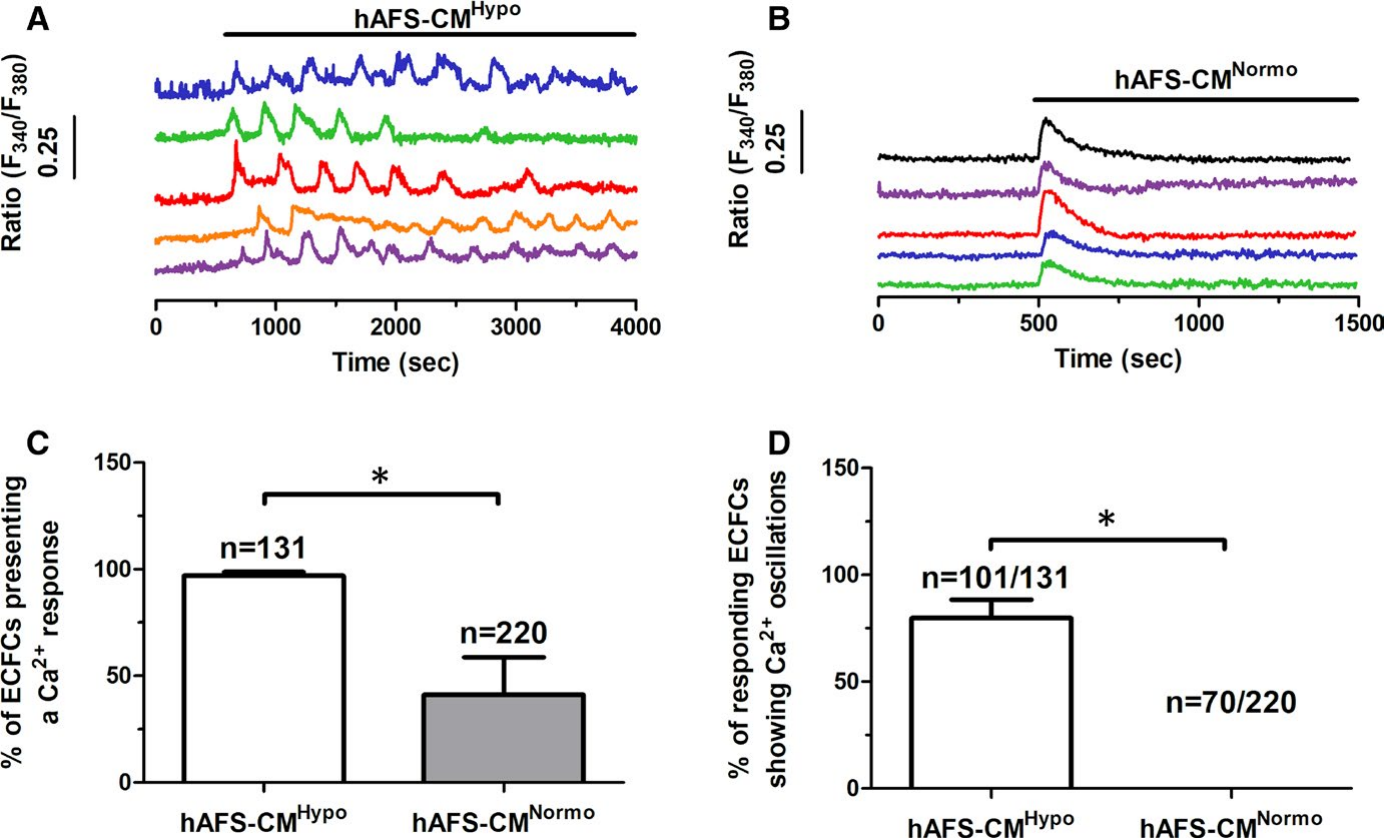
The hypoxic human amniotic fluid‐derived stem cell (hAFS) secretome triggers intracellular Ca^2+^ oscillations in ECFCs. A, intracellular Ca^2+^ oscillations induced by hypoxic hAFS secretome (hAFS‐CM^Hypo^) in circulating endothelial colony‐forming cells (ECFCs) from the same coverslip. B, transient intracellular Ca^2+^ signals evoked by normoxic hAFS secretome (hAFS‐CM^Normo^) in the same population of circulating ECFCs, plated on a different coverslip. The horizontal bar above the Ca^2+^ tracings indicates when Hypo (A) and Normo (B) hAFS secretomes were applied. C, mean ± SE of the percentage of cells displaying a Ca^2+^ response to the different treatments. D, mean ± SE of the percentage of responding cells displaying intracellular Ca^2+^ oscillations (ie more than one Ca^2+^ transient) in response to the different treatments. * indicates *P* < .05 (Student's *t* test)

**FIGURE 2:**
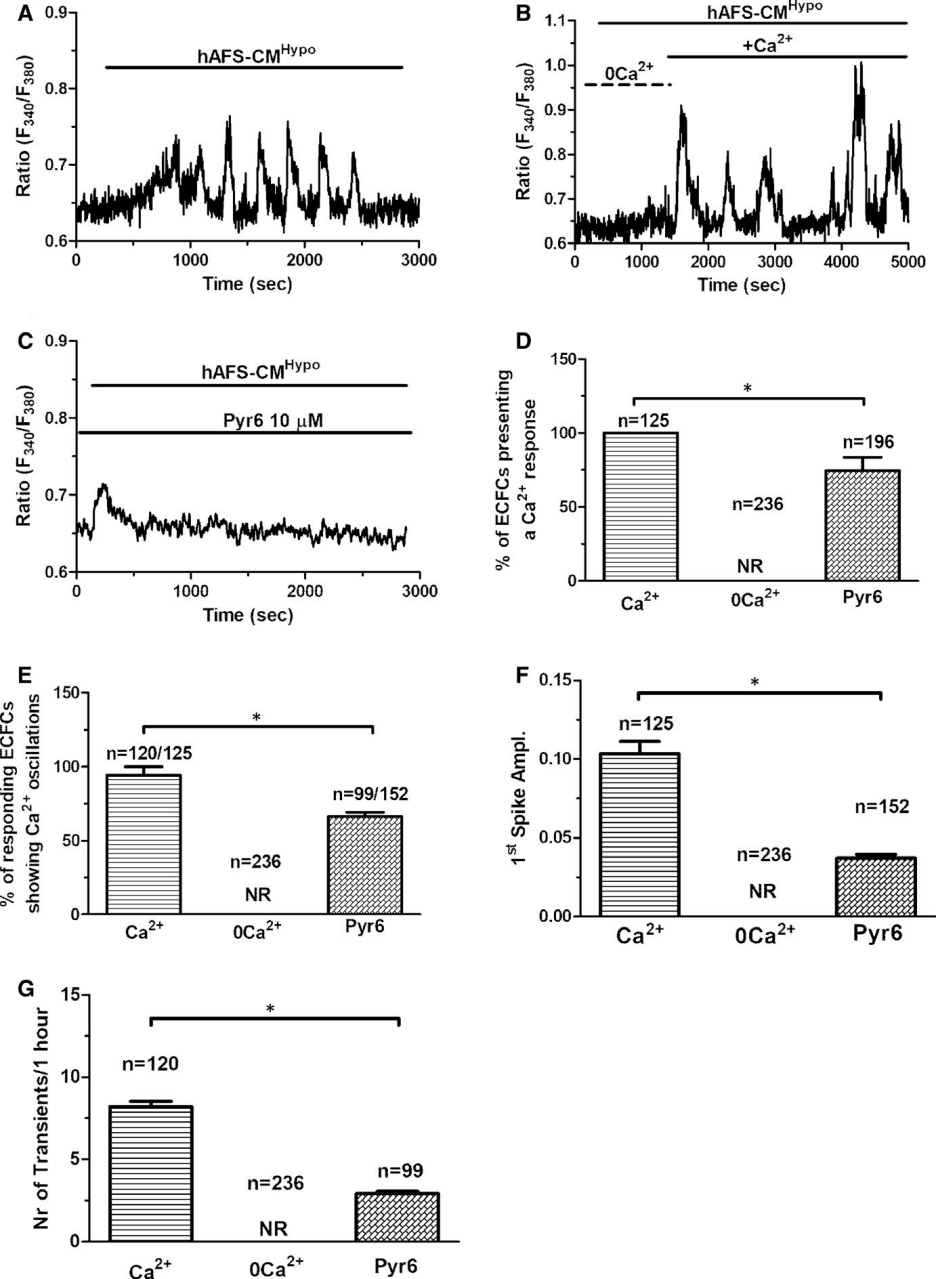
Extracellular Ca^2+^ entry triggers hypoxic human amniotic fluid‐derived stem cell (hAFS) secretome‐induced intracellular Ca^2+^ oscillations in circulating endothelial colony‐forming cells (ECFCs). A, intracellular Ca^2+^ oscillations induced by hypoxic hAFS secretome (hAFS‐CM^Hypo^) in the presence of external Ca^2+^. The horizontal bar above the Ca^2+^ tracings indicates the application period of hAFS‐CM^Hypo^. B, extracellular Ca^2+^ was removed (0 Ca^2+^) at 200 s, and hAFS‐CM^Hypo^ was applied at 300 s from the beginning of the recording. Intracellular Ca^2+^ oscillations were not recorded under 0Ca^2+^ conditions. Extracellular Ca^2+^ was restored at 1500 s, thereby resuming the spiking Ca^2+^ response. Horizontal bars above the Ca^2+^ tracings indicate the application period of hAFS‐CM^Hypo^ and of physiological salt solution (PSS) supplemented (Ca^2+^) or not (0 Ca^2+^) with Ca^2+^. C, 10‐min pre‐incubation with Pyr6 (10 μmol/L), a selective inhibitor of store‐operated Ca^2+^ entry (SOCE), curtailed, but did not prevent, the onset of the Ca^2+^ response to hAFS‐CM^Hypo^. The horizontal bars above the Ca^2+^ tracings indicate the application period of hAFS‐CM^Hypo^ and Pyr6. D, mean ± SE of the percentage of cells displaying a Ca^2+^ response to hAFS‐CM^Hypo^ under the designated treatments. E, mean ± SE of the percentage of cells displaying intracellular Ca^2+^ oscillations (ie more than one Ca^2+^ transient) in response to hAFS‐CM^Hypo^ under the designated treatments. F, mean ± SE of the amplitude of the 1st Ca^2+^ spike elicited by hAFS‐CM^Hypo^ under the designated treatments. G, mean ± SE of the intracellular Ca^2+^ transients elicited by hAFS‐CM^Hypo^ under the designated treatments. * indicates *P* < .05 (Student's *t* test). NR, no response

Human amniotic fluid‐derived stem cells medium conditioned under hypoxia (hAFS‐CM^Hypo^) (Figure 1A), but not under normoxia (hAFS‐CM^Normo^) (Figure 1B), immediately induced repetitive oscillations in [Ca^2+^]_i_ in circulating ECFCs loaded with the Ca^2+^‐sensitive dye Fura‐2/AM (4 µmol/L), thereby confirming the findings recently reported in.^8^, ^9^ Furthermore, the percentage of ECFCs displaying a Ca^2+^ signal was significantly (*P* < .05) larger when hAFS‐CM^Hypo^ was administered (Figure 1C,D). The frequency of the intracellular Ca^2+^ spikes arising during 1‐hour recording ranged between 5 and 11 oscillations/hour and averaged 8.8 ± 1.1 oscillations/hour (n = 101). The endothelial Ca^2+^ response to extracellular stimuli impinges on two Ca^2+^ sources: the extracellular milieu and the endogenous Ca^2+^ stores located within ER cisternae and EL vesicles.^24^, ^25^ hAFS‐CM^Hypo^ induced intracellular Ca^2+^ oscillations in the presence (Figure 2A), but not in the absence (Figure 2B), of extracellular Ca^2+^. However, the spiking Ca^2+^ signal promptly resumed upon Ca^2+^ restitution to the recording solution (Figure 2B). Therefore, extracellular Ca^2+^ entry was required to trigger hAFS‐CM^Hypo^‐induced intracellular Ca^2+^ oscillations. SOCE, which can be recruited by a spatially restricted InsP_3_‐induced ER Ca^2+^ pulse undetectable by epifluorescence imaging,^26^, ^27^ represents the main Ca^2+^‐entry pathway in circulating ECFCs.^28^ The pyrazole derivative BTP‐2 has been shown to specifically inhibit SOCE in ECFCs.^29^, ^30^ Pre‐treating the cells with BTP‐2 (10 µmol/L, 20 minutes) did not prevent the onset of the Ca^2+^ response to hAFS‐CM^Hypo^ (Figure 2C,D), but significantly (*P* < .05) reduced the percentage of oscillating cells (Figure 2E) and the amplitude of the 1st Ca^2+^ spike (Figure 2F). Furthermore, BTP‐2 curtailed the frequency of the Ca^2+^ transients to 1‐2 oscillations/h (Figure 2G). These data demonstrate that SOCE is required to maintain the oscillations over time but is not responsible for the onset of the Ca^2+^ response to hAFS‐CM^Hypo^. Therefore, a store‐independent Ca^2+^‐permeable route initiates the oscillatory signal recorded in the presence of extracellular Ca^2+^, as previously shown in UCB‐derived ECFCs stimulated with VEGF.^13^

### 3.2 hAFS‐CM^Hypo^‐induced intracellular Ca^2+^ oscillations are triggered by TRP Vanilloid 4 (TRPV4) and shaped by InsP_3_ receptors (InsP_3_Rs) and TPC1

**FIGURE 3:**
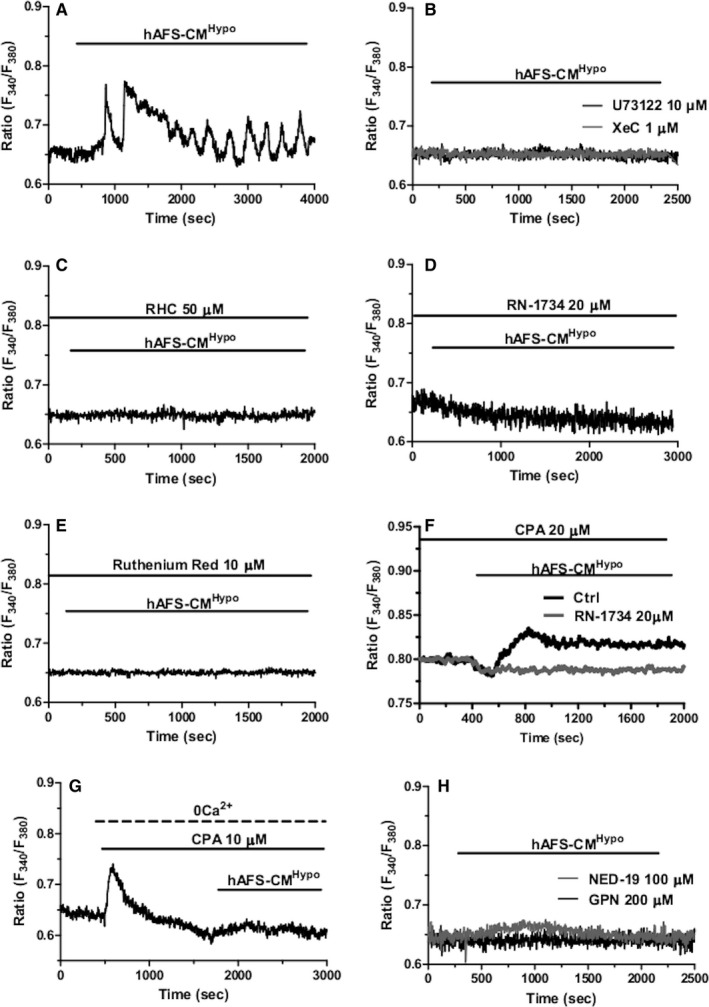
The role of transient receptor potential vanilloid 4 (TRPV4), InsP_3_ receptors (InsP_3_Rs) and two‐pore channel 1 (TPC1) in hypoxic human amniotic fluid‐derived stem cell (hAFS) (hAFS) secretome‐induced intracellular Ca^2+^ oscillations in circulating endothelial colony‐forming cells (ECFCs). A, intracellular Ca^2+^ oscillations induced by the hypoxic hAFS secretome (hAFS‐CM^Hypo^) in circulating ECFCs under control conditions, that is in the presence of extracellular Ca^2+^ and in the absence of inhibitors of the Ca^2+^ signalosome. The horizontal bar above the Ca^2+^ tracings indicates the application period of hAFS‐CM^Hypo^. B, 30‐min pre‐incubation with U73122 (10 μmol/L), an antagonist of phospholipase C (PLC), and 10‐min pre‐incubation with Xestospongin C (XeC; 1 μmol/L, 10 min), a blocker of InsP_3_Rs, prevented the oscillatory response to hAFS secretome. The horizontal bar above the Ca^2+^ tracings indicates the application period of hAFS‐CM^Hypo^. C, 10‐min pre‐incubation with RHC‐80267 (RHC; 50 μmol/L), which selectively interferes with diacylglycerol (DAG) lipase activity, suppressed hAFS‐CM^Hypo^‐induced intracellular Ca^2+^ oscillations in circulating ECFCs. The horizontal bars above the Ca^2+^ tracings indicate the application period of hAFS‐CM^Hypo^ and RHC. D, 30‐min pre‐incubation with RN‐1734 (20 μmol/L), a selective TRPV4 blocker, prevented the oscillatory Ca^2+^ response to hAFS‐CM^Hypo^ in circulating ECFCs. The horizontal bars above the Ca^2+^ tracings indicate the application period of hAFS‐CM^Hypo^ and RN‐1734. E, 10‐min pre‐incubation with ruthenium red (10 μmol/L), a pan‐specific inhibitor of TRPV channels, also suppressed hAFS‐CM^Hypo^‐induced intracellular Ca^2+^ oscillations in circulating ECFCs. The horizontal bars above the Ca^2+^ tracings indicate the application period of hAFS‐CM^Hypo^ and ruthenium red. F, ECFCs were pretreated for 30 min with cyclopiazonic acid (CPA; 10 μmol/L) to fully deplete the endoplasmic reticulum (ER) Ca^2+^ reservoir and activate store‐operated Ca^2+^ entry (SOCE). Thereafter, hAFS‐CM^Hypo^ was added and caused a transient reduction in intracellular Ca^2+^ levels followed by a sustained increase in [Ca^2+^]_i_. 30‐min pre‐incubation with RN‐1734 (20 μmol/L) to block TRPV4 prevented this [Ca^2+^]_i_ rise and unmasked the progressive decrease in Fura‐2 fluorescence, which reflects AA‐dependent SOCE inhibition (please, see the text for a wider explanation). The horizontal bars above the Ca^2+^ tracings indicate the application period of hAFS‐CM^Hypo^ and CPA. G, 30‐min pre‐incubation with NED‐19 (100 µmol/L), a selective two‐pore channel (TPC) blocker, and 30‐min pre‐incubation with the lysosomotropic agent, glycyl‐l‐phenylalanine 2‐naphthylamide (GPN; 200 µmol/L), inhibited the oscillatory Ca^2+^ response to hAFS‐CM^Hypo^. The horizontal bar above the Ca^2+^ tracings indicates the application period of hAFS‐CM^Hypo^

**FIGURE 4:**
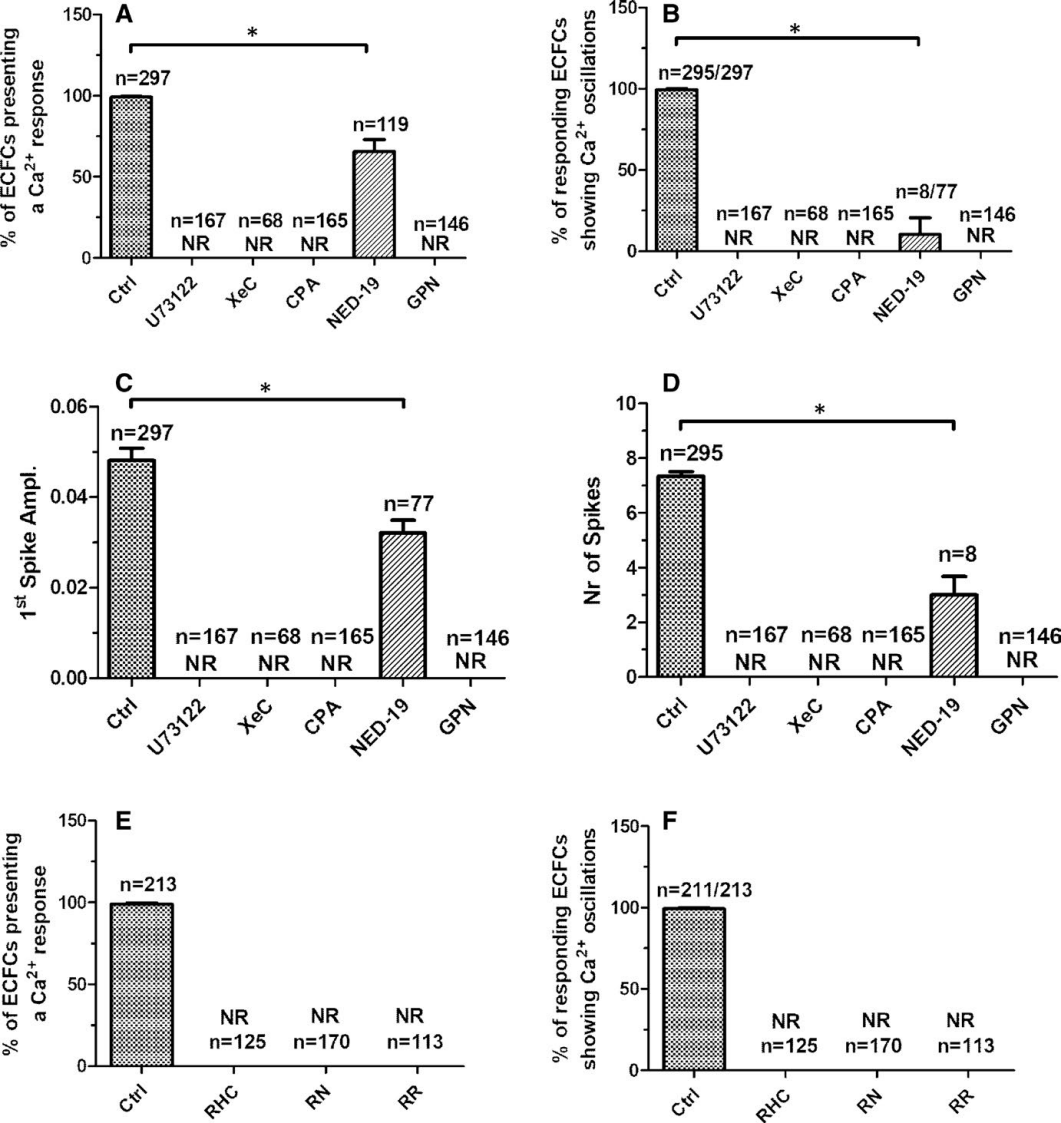
Statistical analysis of phospholipase C (PLC) and nicotinic acid adenine dinucleotide phosphate (NAADP) signalling. Mean ± SE of the percentage of endothelial colony‐forming cells (ECFCs) presenting a Ca^2+^ response (A) and, among these, of ECFCs presenting intracellular Ca^2+^ oscillations (ie more than one Ca^2+^ transient) (B) upon exposure to the hypoxic human amniotic fluid‐derived stem cell (hAFS) secretome (hAFS‐CM^Hypo^) under the designated treatments. Mean ± SE of the amplitude of the 1st Ca^2+^ spike (C) and of the intracellular Ca^2+^ transients (D) elicited by hAFS‐CM^Hypo^ under the designated treatments. Mean ± SE of the percentage of ECFCs presenting a Ca^2+^ response (E) and, among these, of ECFCs presenting intracellular Ca^2+^ oscillations (ie more than one Ca^2+^ transient) (F) when exposed to hAFS‐CM^Hypo^ in the absence (Ctrl) and presence of blockers of transient receptor potential vanilloid 4 (TRPV4) signalling. * indicates *P* < .05 (Student's *t* test). NR, no response

Phospholipase C (PLC) plays a crucial role in the onset of the Ca^2+^ response to chemical stimulation in circulating ECFCs and vascular endothelial cells.^28^ PLC cleaves the minor membrane phospholipid, phosphatidylinositol 4,5‐bisphosphate (PIP_2_), to generate InsP_3_ and DAG. InsP_3_ induces ER Ca^2+^ release by priming InsP_3_Rs to be activated by cytosolic Ca^2+^,^12^, ^19^ whereas DAG may be converted by DAG lipase into arachidonic acid (AA), which in turn activates TRPV4.^31^ The pharmacological blockade of PLC with the aminosteroid U73122 (10 µmol/L, 20 minutes) abrogated hAFS‐CM^Hypo^‐induced intracellular Ca^2+^ oscillations (Figure 3A,B). The same inhibitory effect was achieved by Xestospongin C (XeC; 1 µmol/L, 10 minutes) (Figure 3B), a selective blocker of InsP_3_Rs,^32^ thereby confirming that InsP_3_ contributes to shape the spiking Ca^2+^ signal. The involvement of DAG was assessed by measuring the Ca^2+^ response to hAFS‐CM^Hypo^ in the presence of RHC‐80267 (50 µmol/L, 10 minutes) or RN‐1734 (20 µmol/L, 10 minutes), which, respectively, inhibit DAG lipase^33^ and TRPV4.^23^ Both drugs suppressed hAFS‐CM^Hypo^‐induced intracellular Ca^2+^ oscillations in circulating ECFCs (Figure 3C,D). Similarly, the spiking Ca^2+^ signal was prevented by pre‐treating the cells with ruthenium red (10 µmol/L, 10 minutes) (Figure 3E), a less specific TRPV4 inhibitor.^34^ Collectively, these data provide the evidence that, upon PLC engagement, DAG gates TRPV4 to mediate the influx of extracellular Ca^2+^ that triggers the rhythmical Ca^2+^‐dependent recruitment of InsP_3_Rs. To further corroborate this model, hAFS‐CM^Hypo^ was administered to circulating ECFCs exposed for 30 minutes to cyclopiazonic acid (CPA; 10 µmol/L), which selectively blocks Sarco‐Endoplasmic Reticulum Ca^2+^‐ATPase (SERCA) activity, in the presence of extracellular Ca^2+^ to deplete the ER Ca^2+^ store and full activate SOCE.^13^, ^31^ Therefore, under these conditions, only second messengers‐operated channels may be recruited by extracellular stimuli to increase the [Ca^2+^]_i_. When hAFS‐CM^Hypo^ was delivered upon 20‐minutes exposure to CPA, it induced a sustained elevation in [Ca^2+^]_i_ (Figure 2F) that was abrogated in the absence of extracellular Ca^2+^ and in the presence of RN‐1734 (Figure 2G). Interestingly, addition of the hAFS‐CM^Hypo^ in the presence of external Ca^2+^ caused a transient reduction in [Ca^2+^]_i_, which preceded the gradual re‐emergence of extracellular Ca^2+^ entry and reflects AA‐dependent inhibition of SOCE.^31^, ^35^ Taken together, these findings endorse the view that TRPV4‐mediated extracellular Ca^2+^ entry is required to initiate InsP_3_‐dependent intracellular Ca^2+^ oscillations. A recent report showed that NAADP‐induced EL Ca^2+^ release through TPC1 supports oscillations in [Ca^2+^]_i_ mediated by InsP_3_Rs in ECFCs.^14^ In agreement with these observations, hAFS‐CM^Hypo^ failed to trigger cytosolic Ca^2+^ signals in the presence of the lysosomotropic compound, GPN (200 µmol/L, 30 minutes),^36^, ^37^ and of NED‐19 (100 µmol/L, 30 minutes), a selective TPC antagonist.^38^, ^39^ The statistical analysis of pharmacological manipulation of hAFS‐CM^Hypo^‐induced intracellular Ca^2+^ oscillations has been reported in Figure 4.

### 3.3 hAFS‐CM^Hypo^ induces the nuclear translocation of NF‐κB in a Ca^2+^‐dependent manner

**FIGURE 5:**
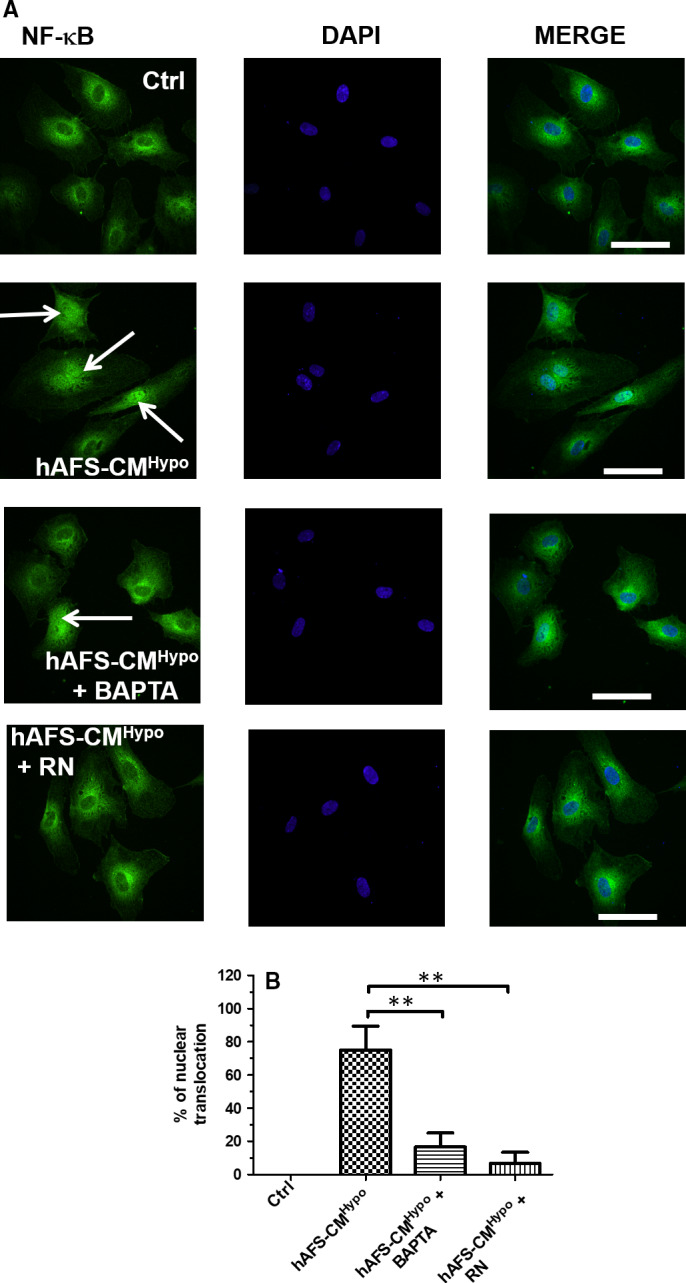
Hypoxic human amniotic fluid‐derived stem cell (hAFS) secretome induces Ca^2+^‐dependent p65 NF‐κB nuclear translocation in circulating endothelial colony‐forming cells (ECFCs). A, immunofluorescence analysis of p65 NF‐κB nuclear translocation in ECFCs untreated (Ctrl) and treated with hypoxic hAFS secretome (hAFS‐CM^Hypo^) for 2 h under control conditions and in the presence of BAPTA (30 µmol/L, 2‐h pre‐incubation before stimulation with hAFS‐CM^Hypo^) or RN‐1734 (RN; 20 μmol/L, 30‐min pre‐incubation before stimulation with hAFS‐CM^Hypo^). The first column shows the fluorescent p65 signal, the second column the nuclei coloured by DAPI (2‐[4‐(Aminoiminomethyl)phenyl]‐1H‐Indole‐6‐carboximidamide hydrochloride), and the third one the merge. B, quantification of nuclear staining of p65 NF‐κB from three coverslips from three independent experiments. Data were shown as mean ± SE. ** indicates *P* < .01 (one‐way ANOVA followed by the post hoc Dunnett's test)

The transcription factor NF‐κB has long been known to translate intracellular Ca^2+^ signals in a pro‐angiogenic output in ECFCs.^12^, ^22^, ^40^ In quiescent cells, the p65 NF‐κB subunit is retained in the cytoplasm by the physical association with the inhibitory IκB protein, but is released from inhibition and primed to translocate into the nucleus by an oscillatory increase in [Ca^2+^]_i_.^15^, ^40^ Immunofluorescence revealed that p65 NF‐κB displayed a cytosolic distribution in non‐stimulated ECFCs (Ctrl; Figure 5A,B), whereas it was mainly accumulated in the nucleus upon exposure to hAFS‐CM^Hypo^ (Figure 5A,B). Conversely, pre‐treating the cells with RN‐1734 (20 µmol/L, 10 minutes), which suppresses the Ca^2+^ spikes, or BAPTA (30 µmol/L, 2 hours), a membrane‐permeable Ca^2+^ buffer,^9^, ^12^ significantly (*P* < .05) inhibited hAFS‐CM^Hypo^‐induced nuclear translocation of p65 NF‐κB (Figure 5A,B). These data, therefore, demonstrate that intracellular Ca^2+^ oscillations drive hAFS‐CM^Hypo^‐induced p65 NF‐κB translocation into the nucleus in circulating ECFCs.

### 3.4 NF‐κB drives hAFS‐CM^Hypo^‐induced ECFC tubulogenesis

**FIGURE 6:**
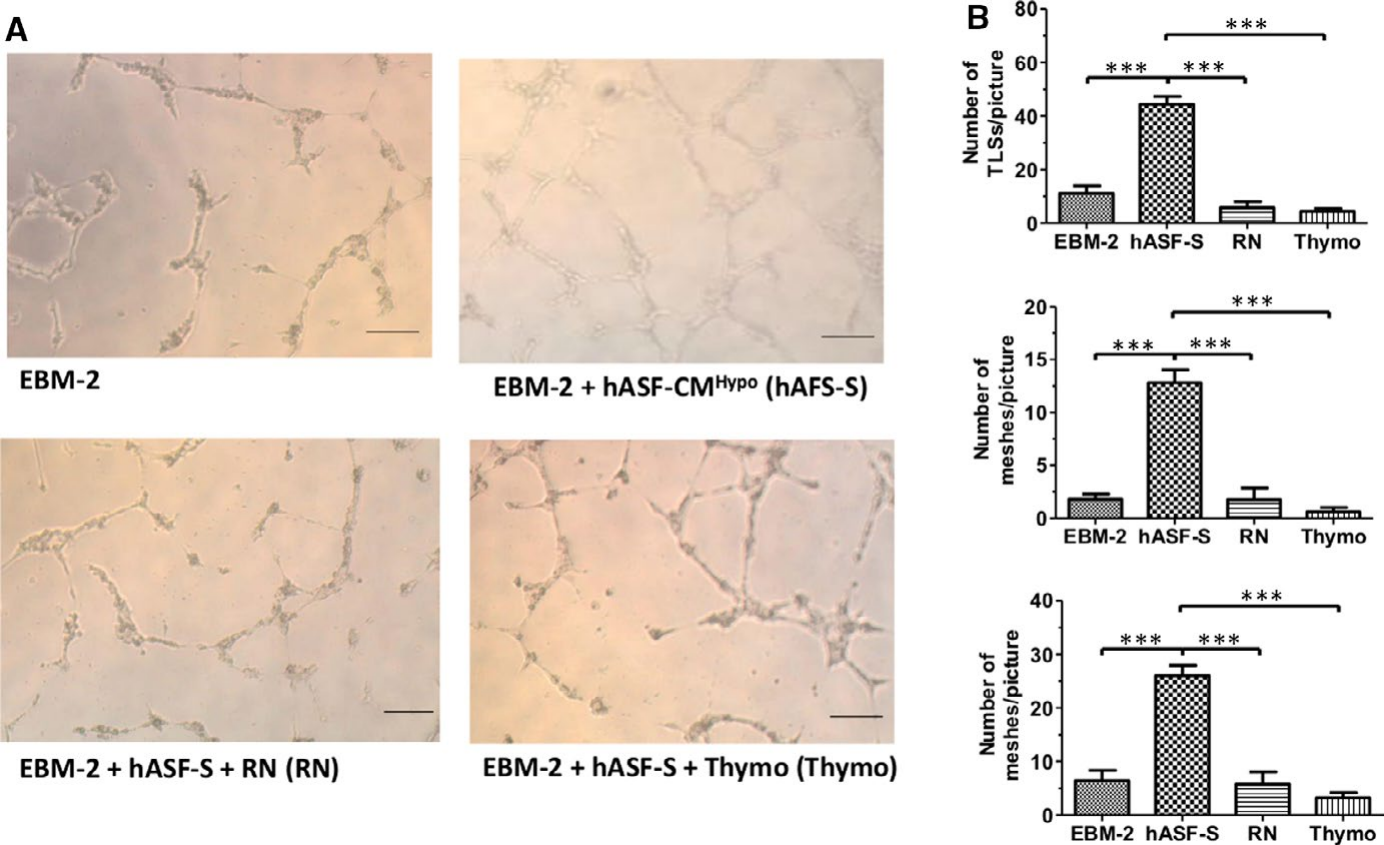
Hypoxic human amniotic fluid‐derived stem cell (hAFS) secretome induces endothelial colony‐forming cell (ECFC) tubulogenesis in Ca^2+^‐ and NF‐κB‐dependent manner. Tubulogenesis assay on ECFCs plated in the presence of EBM‐2 supplemented with 2% foetal bovine serum (FBS) and treated with or without 80 µg/mL of the secretome obtained from hAFS after hypoxic conditioning (hAFS‐CM^Hypo^) supplemented or not with RN‐1734 (RN; 20 μmol/L, 30‐min pre‐incubation before stimulation) or thymoquinone (25 µmol/L, 10‐min pre‐incubation before stimulation). Digital images of endothelial tubes were obtained by bright‐field light microscopy 10 h after plating cells on Matrigel‐coated wells; scale bar: 50 µm. B, mean ± SE of the following parameters evaluated from digital images: number of master tubules (TLSs)/picture (Ba), number of meshes/picture (Bb), number of master junctions/picture (Bc). *** indicate *P* < .0001 (one‐way ANOVA followed by the post hoc Dunnett's test). hAFS‐CM^Hypo^, RN and thymoquinone were maintained during the tubulogenic assay

A recent report from our group demonstrated that hAFS‐CM collected under hypoxic conditions specifically induced ECFC tubulogenesis in vitro.^8^, ^9^ Unlike other in vitro assays, for example migration and invasion, the Matrigel‐based tube formation assay involves all the main physiological steps of the angiogenic process, including endothelial cell proliferation, adhesion, migration and differentiation.^41^ We, therefore, evaluated both topologic (number of meshes and junctions per picture) and dimensional (total number of tubules per picture) of the capillary‐like networks formed by circulating ECFCs placed in a Matrigel scaffold, as shown elsewhere.^19^, ^21^ Preventing the oscillatory increase in [Ca^2+^]_i_ with BAPTA has previously been shown to interfere with hAFS‐CM^Hypo^‐induced ECFC assembly in a bidimensional tubular network.^8^, ^9^ Likewise, ECFCs did not originate capillary tube‐like structures when stimulated with hAFS‐CM^Hypo^ in the presence of RN‐1734 (20 µmol/L, 10 minutes), which prevents the onset of the intracellular Ca^2+^ oscillations, and of thymoquinone (25 µmol/L, 10 minutes) (Figure 6), a selective NF‐κB blocker.^12^, ^42^ Collectively, these data show that hAFS‐CM^Hypo^ requires TRPV4‐mediated extracellular Ca^2+^ entry to trigger the nuclear translocation of p65 NF‐κB and promote ECFC tubulogenesis.

## 4 DISCUSSION

Paracrine therapy through stem cells‐secreted mediators is emerging as an alternative, promising strategy to treat AMI by instructing resident cells, for example cardiac stromal cells, cardiomyocytes and endothelial cells, to optimize endogenous mechanism of cardiac repair.^10^, ^43^ Local delivery of different cell‐conditioned media halted maladaptive remodelling and improved cardiac performance in murine models of AMI. Paracrine signalling was shown to act by restoring cell‐cycle activity or by inhibiting apoptosis/senescence in adult cardiomyocytes and by stimulating angiogenesis in coronary microvascular endothelial cells.^20^, ^44^, ^45^ A recent study by Balbi et al provided a paracrine therapy proof‐of‐principle by demonstrating that single administration via intramyocardial injection of the hAFS secretome in the form of the cell‐conditioned medium reduced infarct area, increased left ventricular ejection fraction, stimulated local angiogenesis and supported cell‐cycle re‐entry of resident surviving cardiomyocytes.^8^, ^9^ In vitro analysis further showed that the hAFS‐CM^Hypo^ stimulated circulating human ECFCs to form capillary‐like networks through an oscillatory increase in [Ca^2+^]_i_.^9^ However, the mechanisms whereby paracrine signalling induces repetitive Ca^2+^ spikes were not dissected. Understanding how the hAFS‐CM^Hypo^ elicits intracellular Ca^2+^ oscillations in ECFCs will provide the biological bases required to decipher the components of the Ca^2+^ toolkit that could be specifically targeted to induce vascular regrowth in infarcted hearts.

### 4.1 hAFS‐CM^Hypo^ induces intracellular Ca^2+^ oscillations by activating TRPV4

Human amniotic fluid‐derived stem cells ‐conditioned medium obtained from human amniotic fluid stem cells maintained under hypoxic over control normoxic conditions induced intracellular Ca^2+^ oscillations in circulating ECFCs, thereby confirming previous results from our group.^9^ Paracrine medium secreted by bone marrow‐derived mesenchymal stem cells cultured under hypoxia was previously shown to dampen mitochondrial Ca^2+^ overload during ischaemia/reperfusion injury.^46^ Thus, paracrine mediators released from stem cells could vary depending on their source and/or their microenvironment and could activate different steps of the complex process of cardiac repair.^43^ The intracellular Ca^2+^ oscillations induced by hAFS‐CM^Hypo^ in circulating ECFCs strongly resembled those induced by VEGF.^12^, ^19^, ^21^ However, hAFS‐CM^Hypo^ does not contain VEGF, whereas it is enriched with multiple cytokines and chemokines, including interleukin 8 (IL‐8), angiogenin, Extracellular matrix metalloproteinase inducer and monocyte chemoattractant protein‐1 (MCP‐1).^47^ Interestingly, an increase in [Ca^2+^]_i_ can be evoked by IL‐8 in mouse lymphokine‐activated killer (LAK) cells^48^ and by MCP‐1 in human monocytes,^49^ although these Ca^2+^ signals do not adopt an oscillatory pattern.

Extracellular stimuli cause an increase in endothelial [Ca^2+^]_i_, as well as in circulating ECFCs,^28^ by promoting extracellular Ca^2+^ entry and/or endogenous Ca^2+^ release.^24^, ^25^, ^50^, ^51^ Removal of extracellular Ca^2+^ prevented the onset of hAFS‐CM^Hypo^‐induced intracellular Ca^2+^ oscillations, which promptly resumed upon restitution of external Ca^2+^. This finding further supports the notion that VEGF is not involved in the spiking Ca^2+^ signal. Indeed, VEGF is still able to trigger 1‐4 intracellular Ca^2+^ spikes in the absence of extracellular Ca^2+^ entry in circulating ECFCs.^12^ Nonetheless, early reports demonstrated that extracellular Ca^2+^ entry through two distinct DAG‐sensitive pathways, that is TRPC1 and TRPC3, induced intracellular Ca^2+^ oscillations, respectively, in primary myelofibrosis‐derived ECFCs^21^ and UCB‐derived ECFCs.^13^ The circulating ECFCs employed in the present investigation do not express TRPC3.^13^ Furthermore, in these cells, TRPC1 is not sensitive to DAG,^21^ but is part of a super‐molecular complex including also Orai1 and STIM1, which is assembled upon depletion of the ER Ca^2+^ store.^28^, ^52^ However, DAG may be converted by DAG lipase in AA,^53^ which selectively gates extracellular Ca^2+^ entry through TRPV4 in circulating ECFCs^31^, ^54^ and vascular endothelial cells.^23^, ^25^ The following pieces of evidence demonstrate that TRPV4‐mediated extracellular Ca^2+^ entry triggers hAFS‐CM^Hypo^‐induced intracellular Ca^2+^ oscillations in circulating ECFCs. First, the pharmacological blockade of TRPV4 with two structurally distinct inhibitors, RN‐1734 and ruthenium red, suppressed the onset of the oscillatory signal. RN‐1734 is a selective TRPV4 antagonist,^34^ and it does not inhibit the other TRPV isoform expressed in circulating ECFCs, that is TRPV1.^22^, ^54^ Second, U73122 and RHC‐80267, which, respectively, inhibit PLC activity^12^ and DAG lipase,^53^ also prevented the oscillatory Ca^2+^ response to hAFS‐CM^Hypo^. Interestingly, the intracellular Ca^2+^ signals evoked by MCP‐1, which is quite abundant in hAFS‐CM,^8^ also require DAG metabolism by DAG lipase in human monocytes.^49^ Third, the pharmacological blockade of Orai1, which contributes to SOCE,^28^, ^52^ curtailed, but did not abrogate, the number of Ca^2+^ transients evoked by hAFS‐CM^Hypo^. Therefore, while SOCE is required to maintain the intracellular Ca^2+^ oscillations by reloading the ER with Ca^2+^ in a SERCA‐dependent manner,^12^, ^15^ it does not ignite the oscillatory Ca^2+^ response.

### 4.2 InsP_3_Rs and TPC1 mediate intracellular Ca^2+^ release during hAFS‐CM^Hypo^‐induced intracellular Ca^2+^ oscillations

InsP_3_Rs provide the main ER Ca^2+^‐releasing pathway that is periodically activated to support intracellular Ca^2+^ oscillations.^15^, ^55^ InsP_3_ is synthesized in response to extracellular stimuli recruiting PLC and sensitizes InsP_3_Rs towards feedback activation by cytosolic Ca^2+^, thereby producing brief Ca^2+^ transients.^13^, ^52^ ECFCs express all the three known InsP_3_R sub‐types, that is InsP_3_R1‐3.^12^ InsP_3_R1 and InsP_3_R2 are especially suitable to support intracellular Ca^2+^ oscillations as they have shown a “bell‐shaped” dependence on surrounding Ca^2+^ ^28^, ^55^: a relatively small increase in local Ca^2+^ concentration activates the InsP_3_‐primed receptors, while the subsequent increase in cytosolic Ca^2+^ (>1 µmol/L) inhibits further Ca^2+^ release. That InsP_3_Rs are required to support hAFS‐CM^Hypo^‐induced intracellular Ca^2+^ oscillations is indicated by the inhibitory effect of U73122 and XeC, which, respectively, target PLC and InsP_3_Rs. Furthermore, depletion of the ER Ca^2+^ store with CPA transformed the repetitive Ca^2+^ transients into a sustained elevation in [Ca^2+^]_i_, which was caused by TRPV4 activation. Therefore, we propose that TRPV4‐mediated extracellular Ca^2+^ entry provides the source of Ca^2+^ that is required to induce the Ca^2+^‐dependent recruitment of InsP_3_Rs by hAFS‐CM^Hypo^. The subsequent InsP_3_‐induced depletion of the ER Ca^2+^ store can, in turn, engage STIM1, a sensor of ER Ca^2+^ concentration, to bind to and gate Orai1 and TRPC1 to mediate SOCE.^28^ NAADP‐induced EL Ca^2+^ release through TPCs may cooperate with InsP_3_Rs to trigger endothelial Ca^2+^ waves.^56^, ^57^ For instance, TPC1 activation is required to trigger VEGF‐induced repetitive Ca^2+^ spikes in circulating ECFCs.^14^ Herein, we provided the evidence that interfering with EL Ca^2+^ release with either GPN or NED‐19, which, respectively, disrupt the EL Ca^2+^ store and inhibit TPC1, also impairs hAFS‐CM^Hypo^‐induced intracellular Ca^2+^ oscillations. Therefore, NAADP‐induced Ca^2+^ release is likely to contribute with TRPV4 to ignite periodical InsP_3_‐induced ER Ca^2+^ release under these circumstances. Interestingly, IL‐8, which is also enriched in hAFS‐CM,^8^ was recently found to stimulate endosomal CD38 to produce NAADP in LAK cells.^48^

### 4.3 hAFS‐CM^Hypo^ promotes in vitro tubulogenesis by inducing the nuclear translocation of NF‐κB

Endothelial Ca^2+^ oscillations support in vitro tubulogenesis and neovessel formation in vivo.^24^ Moreover, we recently demonstrated that preventing the oscillatory increase in [Ca^2+^]_i_ with BAPTA‐AM impaired hAFS‐CM^Hypo^‐induced ECFC tubulogenesis in Matrigel scaffolds. The Ca^2+^‐sensitive transcription factor, NF‐κB, may translate intracellular Ca^2+^ signals into a pro‐angiogenic input in circulating ECFCs. For instance, NF‐κB decodes VEGF‐induced intracellular Ca^2+^ oscillations in ECFCs^12^ and is activated by TRPV1‐mediated extracellular Ca^2+^ entry to induce proliferation and tube formation upon optical excitation of the photosensitive conjugated polymer, P3HT.^22^, ^50^ Furthermore, hAFS‐CM^Hypo^ promotes the nuclear translocation of NF‐κB to counteract doxorubicin‐induced cardiotoxicity in murine neonatal ventricular myocytes.^20^ In agreement with these observations, immunofluorescence revealed that nuclear accumulation of the p65 subunit of the NF‐κB complex was impaired by suppressing intracellular Ca^2+^ oscillations with either RN‐1734 (to block the triggering TRPV4‐mediated Ca^2+^ signal) or BAPTA‐AM (to buffer intracellular Ca^2+^ levels). Of note, repetitive increases in [Ca^2+^]_i_ represent the most suitable Ca^2+^ waveform to recruit NF‐κB.^15^, ^58^ Furthermore, the frequency of hAFS‐CM^Hypo^‐induced intracellular Ca^2+^ oscillations, that is ~9 oscillations/hour or 2.5 mHz, is within the same range as that required to efficiently activate NF‐κB, that is 0.56‐10 mHz.^58^ Of note, preventing the intracellular Ca^2+^ oscillations with RN‐1734 and the downstream recruitment of NF‐κB with thymoquinone potently inhibited hAFS‐CM^Hypo^‐induced ECFC tube formation. This finding is strongly supported by the evidence that multiple pro‐angiogenic genes, for example intercellular adhesion molecule 1, selectin E and various matrix metalloproteinases, are expressed upon the Ca^2+^‐dependent activation of NF‐κB in circulating ECFCs.^22^, ^59^ Furthermore, NF‐κB regulates the expression of a large array of pro‐angiogenic genes, for example those encoding for growth factors (eg VEGF), chemokines and cell adhesion molecules.^59^ Thus, the Ca^2+^‐dependent engagement of NF‐κB is likely to play a crucial role in hAFS‐CM^Hypo^‐induced revascularization in murine models of AMI^8^ and hindlimb ischaemia.^11^ The involvement of other pro‐angiogenic signalling pathways, such as phosphoinositide 3‐kinase/Akt, which can also be activated by hAFS‐CM^Hypo^ ^20^ and is sensitive to Ca^2+^ in ECFCs,^18^ cannot be ruled out.

## 5 CONCLUSIONS

**FIGURE 7:**
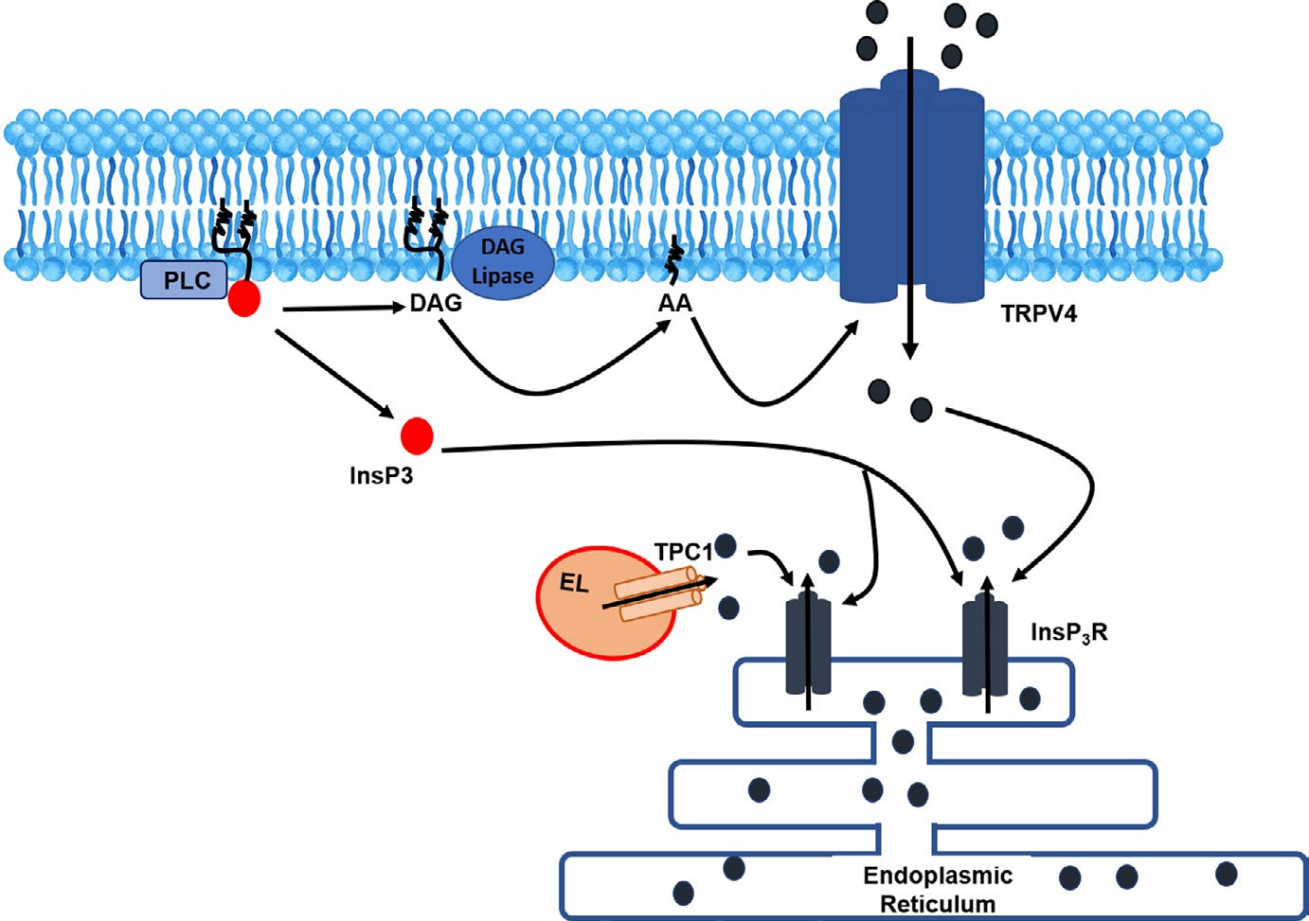
The mechanisms leading to the onset of hypoxic human amniotic fluid‐derived stem cell (hAFS) secretome‐induced intracellular Ca^2+^ oscillations in endothelial colony‐forming cells (ECFCs). Exposure of circulating ECFCs to hypoxic hAFS secretome (hAFS‐CM^Hypo^) results in phospholipase C (PLC) engagement, followed by production of diacylglycerol (DAG) and inositol‐1,4,5‐trisphosphate (InsP_3_). DAG is converted by DAG lipase into arachidonic acid (AA), which gates transient receptor potential vanilloid 4 (TRPV4) to mediate extracellular Ca^2+^ entry through the plasma membrane. InsP_3_ primes ER‐embedded to be activated by the incoming Ca^2+^. Nicotinic acid adenine dinucleotide phosphate (NAADP)‐induced endolysosomal (EL) Ca^2+^ release mediated by two‐pore channel 1 (TPC1) is also likely to contribute to the Ca^2+^‐dependent recruitment of InsP_3_ receptors (InsP_3_Rs). Endoplasmic reticulum (ER) Ca^2+^ depletion, in turn, leads to store‐operated Ca^2+^ entry (SOCE) activation and maintenance of intracellular Ca^2+^ oscillations over time (not shown)

The present investigation reveals for the first time the signalling pathways whereby hAFS‐CM^Hypo^ induces pro‐angiogenic Ca^2+^ oscillations in circulating ECFCs, which represent the most suitable cellular substrate to achieve therapeutic angiogenesis in ischaemic disorders. hAFS‐CM^Hypo^ promotes TRPV4‐mediated extracellular Ca^2+^ entry, which thereby results in InsP_3_‐dependent periodical ER Ca^2+^ release accompanied by SOCE activation (Figure 7). Intracellular Ca^2+^ transients are also supported by NAADP‐induced EL Ca^2+^ release through TPC1, which favours the Ca^2+^‐dependent recruitment of InsP_3_Rs (Figure 7). These findings endorse the emerging view that TRPV4, InsP_3_Rs, TPC1 and SOCE may be targeted through genetic or pharmacological manipulation to enhance the therapeutic outcome of ECFCs‐based therapy.^2^, ^60^ For instance, autologous ECFCs could be genetically manipulated to overexpress TRPV4, thereby boosting their vasoreparative potential in ischaemic disorders. Alternately, specific TRPV4 agonists, such as GSK1016790A,^23^ could be injected into the infarcted myocardium, to boost proliferation and tube formation in ECFCs recruited towards the damaged tissue by the ischaemic insult. Furthermore, they extend at molecular level our knowledge of the mechanisms whereby paracrine therapy through hAFS secretome formulations may induce significant vascular regrowth in widespread ischaemic disorders, such as AMI and hindlimb ischaemia. In the light of such evidence, profiling the components of the hAFS‐CM^Hypo^ that induce these pro‐angiogenic Ca^2+^ oscillations could lead to the formulation of a more efficient cocktail of bioactive factors, which bear the potential to be locally delivered to the ischaemic heart, thereby replacing the time‐consuming and costly cell‐based therapy. This task is currently under way in our laboratories. Due to their ability to induce intracellular Ca^2+^ signals in other cell types, we are assessing whether IL‐8^48^ and MCP‐1^49^ elicit intracellular Ca^2+^ oscillations in ECFCs either alone or in combination. If effective at inducing pro‐angiogenic Ca^2+^ signals, these mediators could be directly injected into the infarcted myocardium to boost ECFCs’ vasoreparative activity.

## CONFLICT OF INTEREST

The authors confirm that there are no conflicts of interest.

## AUTHOR CONTRIBUTIONS

**Valentina Balducci:** Formal analysis (lead); Investigation (lead); Methodology (equal); Writing‐review & editing (supporting). **Pawan Faris:** Formal analysis (supporting). **Carolina Balbi:** Investigation (equal). **Ambra Costa:** Investigation (equal). **Sharon Negri:** Formal analysis (equal). **Vittorio Rosti:** Conceptualization (equal); Investigation (equal); Validation (equal); Writing‐review & editing (equal). **Sveva Bollini:** Conceptualization (equal); Funding acquisition (equal); Investigation (equal); Supervision (equal); Validation (equal); Writing‐review & editing (equal). **Francesco Moccia:** Conceptualization (lead); Funding acquisition (lead); Project administration (lead); Supervision (lead); Validation (lead); Writing‐original draft (lead).

## Supporting information

Supplementary Information

## Data Availability

All the data are fully available upon reasonable request.
