# Supplementary material for: The human amniotic fluid stem cell secretome triggers intracellular Ca2+ oscillations, NF‐κB nuclear translocation and tube formation in human endothelial colony‐forming cells
Source: J Cell Mol Med. 2021 Jul 20;25(16):8074–86. doi: 10.1111/jcmm.16739 (PMC8358861; doi:10.1111/jcmm.16739)
Supplement: Supplementary file 1 — Supplementary Information [file JCMM-25-8074-s001.docx]

**Supplementary Information**

**Isolation and cultivation of ECFCs**

Blood samples (40 ml) collected in EDTA (ethylenediaminetetraacetic acid)-containing tubes were obtained from healthy human volunteers aged from 22 to 28 years old. The Institutional Review Board at “Istituto di Ricovero e Cura a Carattere Scientifico Policlinico San Matteo Foundation” in Pavia approved all protocols. Informed written consent was obtained according to the Declaration of Helsinki of 1975 as revised in 2008. To isolate ECFCs, mononuclear cells (MNCs) were separated from peripheral blood by density gradient centrifugation on lymphocyte separation medium for 30 min at 400g and washed twice in endothelial basal medium-2 (EBM-2) with 2% foetal bovine serum (FBS). A median of 36 x 10^6^ MNCs (range 18-66) were plated on collagen-coated culture dishes (BD Biosciences) in the presence of the endothelial cell growth medium EGM-2 MV Bullet Kit (Lonza) containing EBM-2, 5% FBS, recombinant human (rh) EGF, rhVEGF, rhFGF-B, rhIGF-1, ascorbic acid, and heparin, and maintained at 37°C in 5% CO_2_ and humidified atmosphere. Discard of non-adherent cells was performed after 2 days; thereafter medium was changed three times a week. The outgrowth of endothelial colonies from adherent MNCs was characterized by the formation of a cluster of cobblestone-appearing cells, resembling endothelial cells. That ECFCs-derived colonies belonged to endothelial lineage was confirmed as described in [^1^](#_ENREF_1)^,^[^2^](#_ENREF_2).

***[Ca^2+^]_i_ measurements***

ECFCs were loaded with 4 µM Fura-2 acetoxymethyl ester (Fura-2/AM; 1 mM stock in dimethyl sulfoxide) in PSS for 1 hour at room temperature. After washing in PSS, the coverslip was fixed to the bottom of a Petri dish and the cells observed by an upright epifluorescence Axiolab microscope (Carl Zeiss, Oberkochen, Germany), usually equipped with a Zeiss ×40 Achroplan objective (water-immersion, 2.0 mm working distance, 0.9 numerical aperture). ECFCs were excited alternately at 340 and 380 nm, and the emitted light was detected at 510 nm. A first neutral density filter (1 or 0.3 optical density) reduced the overall intensity of the excitation light and a second neutral density filter (optical density=0.3) was coupled to the 380 nm filter to approach the intensity of the 340 nm light. A round diaphragm was used to increase the contrast. The excitation filters were mounted on a filter wheel (Lambda 10, Sutter Instrument, Novato, CA, USA). Custom software, working in the LINUX environment, was used to drive the camera (Extended-ISIS Camera, Photonic Science, Millham, UK) and the filter wheel, and to measure and plot on-line the fluorescence from 10 up to100 rectangular “regions of interest” (ROI). Each ROI was identified by a number. Since cell borders were not clearly identifiable, a ROI may not include the whole cell or may include part of an adjacent cell. Adjacent ROIs never superimposed. [Ca^2+^]_i_ was monitored by measuring, for each ROI, the ratio of the mean fluorescence emitted at 510 nm when exciting alternatively at 340 and 380 nm (shortly termed "ratio"). An increase in [Ca^2+^]_i_ causes an increase in the ratio [^3^](#_ENREF_3). Ratio measurements were performed and plotted on-line every 3 s. The experiments were performed at room temperature (22°C).

1. Zuccolo E, Di Buduo C, Lodola F, et al. Stromal Cell-Derived Factor-1alpha Promotes Endothelial Colony-Forming Cell Migration Through the Ca(2+)-Dependent Activation of the Extracellular Signal-Regulated Kinase 1/2 and Phosphoinositide 3-Kinase/AKT Pathways. *Stem Cells Dev*. Jan 1 2018;27(1):23-34. https://doi.org10.1089/scd.2017.0114.

2. Lodola F, Laforenza U, Cattaneo F, et al. VEGF-induced intracellular Ca^2+^ oscillations are down-regulated and do not stimulate angiogenesis in breast cancer-derived endothelial colony forming cells. *Oncotarget*. 2017;8:95223-95246. https://doi.orghttps://doi.org/10.18632/oncotarget.20255.

3. Sanchez-Hernandez Y, Laforenza U, Bonetti E, et al. Store-operated Ca(2+) entry is expressed in human endothelial progenitor cells. *Stem Cells Dev*. Dec 2010;19(12):1967-81. https://doi.org10.1089/scd.2010.0047.
